# Supplementary material for: Identifying Ovarian Cancer in Symptomatic Women: A Systematic Review of Clinical Tools
Source: Cancers (Basel). 2020 Dec 8;12(12):3686. doi: 10.3390/cancers12123686 (PMC7764009; doi:10.3390/cancers12123686)
Supplement: Supplementary file 1 [file cancers-12-03686-s001.pdf]

cancers

Supplemental Materials

Identifying Ovarian Cancer in Symptomatic Women: A Systematic Review of Clinical Tools

Garth Funston, Victoria Hardy, Gary Abel, Emma J. Crosbie, Jon Emery, Willie Hamilton and Fiona M. Walter

Table S1. PRISMA checklist.

Section/topic	#	Checklist item	Location
		**TITLE**	
Title	1	Identify the report as a systematic review, meta-analysis, or both.	Title
		**ABSTRACT**	
Structured summary	2	Provide a structured summary including, as applicable: background; objectives; data sources; study eligibility criteria, participants, and interventions; study appraisal and synthesis methods; results; limitations; conclusions and implications of key findings; systematic review registration number.	Simple summary + Abstract
		**INTRODUCTION**	
Rationale	3	Describe the rationale for the review in the context of what is already known.	Intro para 1 +2
Objectives	4	Provide an explicit statement of questions being addressed with reference to participants, interventions, comparisons, outcomes, and study design (PICOS).	Intro para 1+ 2
		**METHODS**	
Protocol and registration	5	Indicate if a review protocol exists, if and where it can be accessed (e.g., Web address), and, if available, provide registration information including registration number.	Methods para 1
Eligibility criteria	6	Specify study characteristics (e.g., PICOS, length of follow-up) and report characteristics (e.g., years considered, language, publication status) used as criteria for eligibility, giving rationale.	Methods para 1 + 2
Information sources	7	Describe all information sources (e.g., databases with dates of coverage, contact with study authors to identify additional studies) in the search and date last searched.	Methods para 1
Search	8	Present full electronic search strategy for at least one database, including any limits used, such that it could be repeated.	S1 Text
Study selection	9	State the process for selecting studies (i.e., screening, eligibility, included in systematic review, and, if applicable, included in the meta-analysis).	Methods para 3
Data collection process	10	Describe method of data extraction from reports (e.g., piloted forms, independently, in duplicate) and any processes for obtaining and confirming data from investigators.	Methods para 4
Data items	11	List and define all variables for which data were sought (e.g., PICOS, funding sources) and any assumptions and simplifications made.	Methods para 4
Risk of bias in individual studies	12	Describe methods used for assessing risk of bias of individual studies (including specification of whether this was done at the study or outcome level), and how this information is to be used in any data synthesis.	Methods para 6
Summary measures	13	State the principal summary measures (e.g., risk ratio, difference in means).	Methods para 5
Synthesis of results	14	Describe the methods of handling data and combining results of studies, if done, including measures of consistency (e.g., I^2) for each meta-analysis.	Methods para 5
Risk of bias across studies	15	Specify any assessment of risk of bias that may affect the cumulative evidence (e.g., publication bias, selective reporting within studies).	N/A
Additional analyses	16	Describe methods of additional analyses (e.g., sensitivity or subgroup analyses, meta-regression), if done, indicating which were pre-specified.	N/A
		**RESULTS**	

Study selection	17	Give numbers of studies screened, assessed for eligibility, and included in the review, with reasons for exclusions at each stage, ideally with a flow diagram.	Results para 1
Study characteristics	18	For each study, present characteristics for which data were extracted (e.g., study size, PICOS, follow-up period) and provide the citations.	Results para 2, Table 1 and 2
Risk of bias within studies	19	Present data on risk of bias of each study and, if available, any outcome level assessment (see item 12).	Results para 3
Results of individual studies	20	For all outcomes considered (benefits or harms), present, for each study: (a) simple summary data for each intervention group (b) effect estimates and confidence intervals, ideally with a forest plot.	Results para 7-12, Table 4
Synthesis of results	21	Present results of each meta-analysis done, including confidence intervals and measures of consistency.	N/A
Risk of bias across studies	22	Present results of any assessment of risk of bias across studies (see Item 15).	N/A
Additional analysis	23	Give results of additional analyses, if done (e.g., sensitivity or subgroup analyses, meta-regression [see Item 16]).	N/A
**DISCUSSION**			
Summary of evidence	24	Summarize the main findings including the strength of evidence for each main outcome; consider their relevance to key groups (e.g., healthcare providers, users, and policy makers).	Discussion para 1-7
Limitations	25	Discuss limitations at study and outcome level (e.g., risk of bias), and at review-level (e.g., incomplete retrieval of identified research, reporting bias).	Discussion para 2
Conclusions	26	Provide a general interpretation of the results in the context of other evidence, and implications for future research.	Conclusions
**FUNDING**			
Funding	27	Describe sources of funding for the systematic review and other support (e.g., supply of data); role of funders for the systematic review.	Funding section

Text S1. MEDLINE search strategy.

1 ((ovar* or fallopian or peritone*) and (cancer* or neoplas* or tumour* or tumor* or malignan*)).ti,ab.
2 exp Ovarian Neoplasms/ or exp Fallopian Tube Neoplasms/ or exp Peritoneal Neoplasms/
3 1 or 2
4 symptom*.ti,ab.
5 exp symptom assessment/
6 4 or 5
7 (risk* or probabilit* or likelihood* or chance*).ti,ab.
8 exp Risk/ or exp Risk factors/ or exp Probability/
9 predict*.ti,ab.
10 exp "Early Detection of Cancer"/
11 diagnos*.ti,ab.
12 7 or 8 or 9 or 10 or 11 (5845128)
13 (model* or algorithm* or tool* or index* or score* or rule*).ti,ab.
14 exp models, statistical/ or exp algorithms/
15 13 or 14
16 3 and 6 and 12 and 15
17 limit 16 to yr="2000 -Current"

Table S2. Specific study exclusions.

Author, Date	Specific exclusions
Lurie, 2009	*Controls*: Hx OC, no intact ovaries
Rossing, 2010	Non-English speakers, no residential telephone<br>*Controls*: Hx OC, no intact ovaries
Jordan, 2010	*Cases:* language difficulties, mental incapacity, illness<br>*Controls:* language difficulties, illness, previous ovarian cancer or previous bilateral oophorectomy [1]
Hamilton, 2009	No entry in the records ≤1 year pre-diagnosis (cases), previous OC or bilateral oophorectomy, lived outside study area at time of diagnosis (cases) [2]
Hippisley-Cox, 2012	Hx bilateral oophorectomy or OC, 'red flag symptom' ≤12 months before study entry date[a], no postcode related Townsend score
Hippisley-Cox, 2013	'Red flag symptom' ≤12 months before study entry date, no postcode-related Townsend score
Grewal, 2013, UK	No entry in the records ≤1 year pre-diagnosis (cases), previous OC or bilateral oophorectomy, lived outside study area at time of diagnosis (cases) [2]
Collins, 2013	As per Hippisley-Cox, 2011
Goff, 2006	Screening control criteria as outlined in OCEDS [3]
Anderson, 2008	Screening control criteria as outlined in OCEDS [3]
Anderson, 2010	Screening control criteria as outlined in OCEDS [3][b]
Lim, 2012	*Controls:* Hx of bilateral oophorectomy or OC, active malignancy, increased risk of familial OC, not post menopause (as per UKCTOCS trial criteria) [4]
Kim, 2009	*Pap smear controls:* Hx of gynaecological malignancy, no intact ovaries or uterus
Macuks, 2011	Severe co-morbidities, previous or other coexisting malignancies
Shetty, 2015	*Controls (gynae check-up group):* no ovaries, no intact uterus
Jain, 2018	*Cases:* Hx of ovarian cancer, Hx bilateral oophorectomy, recall difficulty, inoperable

[a] Loss of appetite, weight loss, abdominal pain, abdominal distension, rectal bleeding, or postmenopausal bleeding; [b] Patients with known BRCA mutations excluded during study.

Table S3. Tool specifications.

Tool	First study (author, year)	Specification
	Symptom checklists	
Goff SI	Goff, 2007	Tool positive if any of pelvic/abdominal pain, increased abdominal size/bloating, and difficulty eating/feeling full occurred >12 times per month but were present for <1 year

Modified Goff SI 1	Kim, 2009	Tool positive if any of pelvic/abdominal pain, urinary urgency/frequency, increased abdominal size/bloating, difficulty eating/feeling full present for <1 year that occurred >12 times per month
Lurie 7-SI	Lurie, 2009	Tool positive if any of distended abdomen (defined as "persistent distended and hard abdomen"), abnormal vaginal bleeding (defined as "vaginal bleeding not associated with periods"), palpable abdominal mass (defined as "a palpable abdominal mass that woman herself had noticed"), abdominal pain (defined as "persistent abdominal or pelvic pain or discomfort"), urinary symptoms (defined as "urinary frequency, difficulty emptying urinary bladder, or dysuria"), bowel symptoms (defined as "unusual bowel irregularity such as diarrhoea or constipation, flatulence, or bloating"), and fatigue/appetite loss (defined as "persistent fatigue or loss of appetite"), present in previous 12 months
Lurie 5-SI	Lurie, 2009	Tool positive if any of distended abdomen (defined as "persistent distended and hard abdomen"), abnormal vaginal bleeding (defined as "vaginal bleeding not associated with periods"), palpable abdominal mass (defined as "a palpable abdominal mass that woman herself had noticed"), abdominal pain (defined as "persistent abdominal or pelvic pain or discomfort"), and urinary symptoms (defined as "urinary frequency, difficulty emptying urinary bladder, or dysuria"), present in previous 12 months
Lurie 4-SI	Lurie, 2009	Tool positive if any of distended abdomen (defined as "persistent distended and hard abdomen"), abnormal vaginal bleeding (defined as "vaginal bleeding not associated with periods"), palpable abdominal mass (defined as "a palpable abdominal mass that woman herself had noticed"), and abdominal pain (defined as "persistent abdominal or pelvic pain or discomfort"), present in previous 12 months
Lurie 3-SI	Lurie, 2009	Tool positive if any of distended abdomen (defined as "persistent distended and hard abdomen"), abnormal vaginal bleeding (defined as "vaginal bleeding not associated with periods"), and palpable abdominal mass (defined as "a palpable abdominal mass that woman herself had noticed"), present in previous 12 months
Hamilton SI	Hamilton, 2009	Tool positive if any of bloating, urinary frequency, rectal bleeding, postmenopausal bleeding, loss of appetite, abdominal pain, abdominal distension, present in the previous 12 months
SGO consensus criteria	Rossing, 2010	Tool positive if any of bloating or feeling full, pelvic or abdominal pain, or urinary urgency or frequency, present for at least 1 month, with an onset of less than 12 months
Lim SI 1	Lim, 2012	Tool positive if any of pelvic/abdominal pain or discomfort, loss of appetite or feeling full quickly, weight loss, increase in abdominal size, abdomen feels bloated, and able to feel a lump in the abdomen in previous 12 months
Lim SI 2	Lim, 2012	Tool positive if any of pelvic abdominal pain or discomfort, loss of appetite, increase in abdominal size, able to feel a lump in the abdomen, and vaginal discharge in previous 12 months
Hippisley-Cox SI	Hippisley-Cox, 2012	Tool positive if currently consulting general practitioner with first onset of any of abdominal pain, abdominal distension, appetite loss, rectal bleeding, postmenopausal bleeding, weight loss.
Modified Goff SI 2	Shetty, 2015	Tool positive if any of abdominal/pelvic pain, increased abdominal size/bloating, difficulty in eating/feeling full and urinary frequency/urgency, loss of appetite/weight, occurred >12 times per month and time since onset was <1 year
	Augmented symptom checklist	
Goff SI + CA125	Anderson, 2008	The threshold for a positive CA125 test was determined by dichotomizing CA 125 at the 95th percentile in the control group (threshold approx. 30 u/ml).
Goff SI + HE4	Anderson, 2010	The threshold for a positive HE4 test was determined by dichotomizing HE4 at the 95th percentile in the control group.
Goff SI + HE4 + CA125	Anderson, 2010	The threshold for a positive HE4 and CA125 tests were determined by dichotomizing HE4 at the 95th percentile in the control group. Study evaluated several thresholds for a positive tool (**Table 4**).
Goff SI + CA125 + menopause	Macuks, 2011	CA125 thresholds: 25 U/ml, 35 U/ml and 65 U/ml examined. Definition of menopause not specified.
	Prediction models	

QCancer Ovarian	Hippisley-Cox, 2012	The prediction model included age, family history of ovarian cancer, haemoglobin <110 g/L in past year, currently consulting general practitioner with first onset of any of abdominal pain, abdominal distension, appetite loss, rectal bleeding, postmenopausal bleeding, weight loss. Tool threshold was set based on risk level e.g. 10% of women at highest risk deemed tool positive.
QCancer Female	Hippisley-Cox, 2013	The prediction model included age, BMI, Townsend score, smoking status, alcohol status, family history of gastrointestinal cancer, family history of breast cancer, family history of ovarian cancer, type 2 diabetes, COPD, endometrial hyperplasia or polyp, chronic pancreatitis. Current: loss of appetite, unintentional weight loss, abdominal pain, abdominal swelling, difficulty swallowing, heartburn or indigestion, rectal bleeding, blood in urine, blood in vomit, blood when cough, postmenopausal bleeding, irregular menstrual bleeding, vaginal bleeding after sex, a breast lump, breast skin tethering or nipple discharge, breast pain, a lump in your neck, night sweats, a venous thromboembolism. In the last year seen GP with: change in bowel habit, constipation, cough, unexplained bruising, anaemia (haemoglobin <11g/dL). Tool threshold was set based on risk level e.g. 10% of women at highest risk deemed tool positive.
OC Scores A	Grewal, 2013	Variables: bloating, urinary frequency, rectal bleeding, postmenopausal bleeding, loss of appetite, abdominal pain, abdominal distension, present in the previous 12 months. Model used conditional logistic logarithmic odds ratio of each symptom, to three significant figures. Various threshold reported (**Table 4**).
OC Scores B	Grewal, 2013	Variables: bloating, urinary frequency, rectal bleeding, postmenopausal bleeding, loss of appetite, abdominal pain, abdominal distension, present in the previous 12 months. Model used the conditional logarithmic odds ratio of each variable rounded to the nearest integer. Various threshold reported (**Table 4**).
OC Scores C	Grewal, 2013	Variables: Age (≥50 years / < 50 years), bloating, urinary frequency, rectal bleeding, postmenopausal bleeding, loss of appetite, abdominal pain, abdominal distension, present in the previous 12 months. Various threshold reported (**Table 4**).

Table S4. Deviations from the original Goff SI in validation studies.

Study	Deviation
Rossing, 2010	1) Symptom criteria listed as "bloating or feeling full", whereas original Goff SI includes "Increased abdominal size/bloating" and "difficulty eating/feeling full". 2) Duration/frequency of symptoms criteria was "present at least daily for at least 1 week", whereas the original Goff SI criteria is >12x/month.
Jordan, 2010	Duration/frequency of symptoms criteria was ">2 weeks in previous 12 months" whereas the original Goff SI criteria is >12x/month.
Lim, 2012	Duration/frequency of symptoms criteria was "occurred 16-31 days per month" for interview and questionnaire study components, and no frequency criteria was applied in the GP notes study component. The original Goff SI criteria is >12x/month.

Reference

1. Merritt, M.A.; Green, A.C.; Nagle, C.M.; Webb, P.M.; Bowtell, D.; Chenevix-Trench, G.; Green, A.; Webb, P.; DeFazio, A.; Gertig, D.; et al. Talcum powder, chronic pelvic inflammation and NSAIDs in relation to risk of epithelial ovarian cancer. *Int. J. Cancer* **2008**, *122*, 170–6, doi:10.1002/ijc.23017.
2. Hamilton, W.; Peters, T.J.; Bankhead, C.; Sharp, D. Risk of ovarian cancer in women with symptoms in primary care: population based case-control study. *BMJ* **2009**, *339*, b2998.
3. Lowe, K.A.; Shah, C.; Wallace, E.; Anderson, G.; Paley, P.; McIntosh, M.; Andersen, M.R.; Scholler, N.; Bergan, L.; Thorpe, J.; et al. Effects of personal characteristics on serum CA125, mesothelin, and HE4 levels in healthy postmenopausal women at high-risk for ovarian cancer. *Cancer Epidemiol. Biomarkers Prev.* **2008**, *17*, 2480–2487, doi:10.1158/1055-9965.EPI-08-0150.
4. Jacobs, I.J.; Menon, U.; Ryan, A.; Gentry-Maharaj, A.; Burnell, M.; Kalsi, J.K.; Amso, N.N.; Apostolidou, S.; Benjamin, E.; Cruickshank, D.; et al. Ovarian cancer screening and mortality in the UK Collaborative Trial of Ovarian Cancer Screening (UKCTOCS): A randomised controlled trial. *Lancet* **2016**, *387*, 945–956.
